# Optimal Combination of Glycine, Asparagine, and Phenylalanine Promotes α-Casein Synthesis and Secretion in MAC-T Cells Through Activation of the PI3K-AKT-mTOR Pathway

**DOI:** 10.3390/ani16132038

**Published:** 2026-07-02

**Authors:** Xinyu Zhang, Yu Ding, Min Yang, Ruoshan Luo, Yang Yang, Hang Zhang, Wanping Ren, Liang Yang, Yong Wei, Yankun Zhao, Tongjun Guo, Wei Shao

**Affiliations:** 1 Xinjiang Key Laboratory of Meat and Milk Production Herbivore Nutrition, College of Animal Science, Xinjiang Agricultural University, Urumqi 830052, China; 18663955139@163.com (X.Z.); 18481902483@163.com (Y.D.); y1375724150@163.com (M.Y.); 17809968072@163.com (R.L.); yy14799670731@163.com (Y.Y.); 17382543605@163.com (H.Z.); rwp15999154824@163.com (W.R.); yangliangagu@sina.com (L.Y.); wy-260@163.com (Y.W.); 2 Agricultural Products Quality and Safety Risk Assessment Laboratory of the Ministry of Agriculture and Rural Affairs (Urumqi) and Xinjiang Agricultural Products Quality and Safety Laboratory, Institute of Agricultural Quality Standards and Testing Technology, Xinjiang Academy of Agricultural Sciences, Xinjiang Uygur Autonomous Region, Urumqi 830052, China; yankunzhao90@xaas.ac.cn; 3 Feed Research Institute, Xinjiang Uygur Autonomous Region Academy of Animal Husbandry Sciences, Urumqi 830052, China; guotaoxj@126.com

**Keywords:** glycine, asparagine, phenylalanine, α-casein, PI3K-AKT-mTOR signaling pathway, MAC-T cells

## Abstract

Efficient milk protein synthesis in dairy cows, particularly casein production, is crucial for milk quality but has low nitrogen conversion efficiency. This study aimed to determine whether an optimal ratio of glycine, asparagine, and phenylalanine could synergistically promote α-casein synthesis in bovine mammary epithelial cells (MAC-T) and to elucidate its mechanism via the PI3K-AKT-mTOR signaling pathway. Single-factor experiments and response surface central composite design were conducted to determine the optimal amino acid combination. α-Casein synthesis was measured by ELISA, gene expression by RT-qPCR, and protein phosphorylation by Western blot. A PI3K-specific inhibitor (LY294002) was used in a blocking experiment to validate the involvement of the PI3K-AKT-mTOR pathway. Results: The optimal ratio was 9.898 mmol/L glycine, 7.014 mmol/L asparagine, and 5.865 mmol/L phenylalanine (molar ratio 1.69:1.20:1.00). This combination significantly increased α-casein synthesis and secretion compared to any single amino acid (*p* < 0.01), demonstrating a synergistic effect. It also upregulated *CSN1S1* and *CSN1S2* expression and activated the PI3K-AKT-mTOR pathway at both transcriptional and translational levels. The addition of LY294002 completely abolished these effects, confirming the pathway’s crucial role. The optimal combination of glycine, asparagine, and phenylalanine synergistically enhances α-casein synthesis in MAC-T cells by activating the PI3K-AKT-mTOR pathway. These findings provide a theoretical basis for developing targeted amino acid supplementation strategies to improve milk protein production in dairy cows.

## Simple Summary

Efficient milk protein synthesis in dairy cows is critical for producing high-quality milk. Milk protein is mainly composed of casein, which is synthesized by mammary epithelial cells. Certain amino acids, the building blocks of proteins, not only serve as raw materials but also act as signals that turn on protein synthesis. This study investigated whether a specific mixture of three amino acids—glycine, asparagine, and phenylalanine—could work together to increase casein production more effectively than any single amino acid alone. Using a laboratory model of bovine mammary epithelial cells, we determined the optimal ratio of these three amino acids: 9.898 mmol/L glycine, 7.014 mmol/L asparagine, and 5.865 mmol/L phenylalanine (molar ratio 1.69:1.20:1.00). This combination significantly increased casein synthesis and secretion, outperforming each amino acid individually. The mixture also activated an intracellular signaling network known as the PI3K-AKT-mTOR pathway, which serves as a master switch for protein production. Blocking this pathway with a specific inhibitor stopped the promoting effect, confirming its key role. These findings provide a scientific basis for developing more precise nutritional strategies to improve milk protein yield in dairy cows, ultimately benefiting both farmers and consumers.

## 1. Introduction

The efficiency of milk protein synthesis in dairy cows directly determines milk quality and economic value. Casein, the major milk protein, is synthesized by mammary epithelial cells and is regulated by both the supply of amino acid substrates and intracellular signaling pathways. In recent years, the role of amino acids has expanded from being simple building blocks to functioning as signaling molecules that regulate milk protein synthesis. The PI3K-AKT-mTOR signaling pathway is a central hub through which amino acids control the transcription and translation of casein genes [1,2].

Glycine, asparagine, and phenylalanine are not only components of α-casein but also possess specific regulatory functions. Glycine promotes the proliferation of bovine mammary epithelial cells, providing a favorable cellular basis for casein synthesis [3], and an appropriate concentration enhances α-casein synthesis in MAC-T cells (a widely used bovine mammary epithelial cell line) [4]. Asparagine contributes to ammonia balance and cell viability [5]. Phenylalanine, an essential amino acid, activates the mTOR signaling pathway and promotes the uptake of multiple amino acids, thereby facilitating milk protein synthesis [6]. Although supplementation with a single amino acid can promote α-casein synthesis, its effect is dose-dependent and often suboptimal [7]. In contrast, supplying multiple amino acids at specific ratios better mimics the physiological amino acid profile and may synergistically enhance casein synthesis. For example, methionine and leucine activate the PI3K-mTOR pathway to promote β-casein synthesis [8], and maintaining an optimal lysine-to-methionine ratio while supplementing other essential amino acids activates the mTORC1 pathway, improving milk protein production [9]. Beyond these traditional limiting amino acids, we are interested in the potential synergistic effects of non-essential and conditionally essential amino acids. Glycine and asparagine are abundant in casein but are not typically considered rate-limiting in lactating cows, whereas phenylalanine serves as both a precursor and an activator of mTOR signaling. We reasoned that the simultaneous presence of these three amino acids, each contributing a distinct function, might produce cooperative effects that surpass the sum of their individual actions.

Therefore, this study used MAC-T cells as an experimental model. First, single-factor experiments were conducted to determine the appropriate concentration ranges of the three amino acids. Then, a response surface central composite design was applied to screen for the optimal ratio of glycine, asparagine, and phenylalanine that promotes α-casein synthesis. The effects of this optimal combination on α-casein gene expression and the PI3K-AKT-mTOR signaling pathway were examined at both transcriptional and translational levels. Furthermore, a blocking experiment using the specific PI3K inhibitor LY294002 was performed to verify the role of this pathway. This study aims to elucidate the molecular mechanism by which the optimal combination of glycine, asparagine, and phenylalanine regulates α-casein synthesis in vitro, providing a mechanistic basis for future nutritional intervention strategies.

## 2. Materials and Methods

### 2.1. Test Materials

L-Glycine, L-Asparagine, L-Phenylalanine, and dimethyl sulfoxide were purchased from Sigma-Aldrich (St. Louis, MO, USA). DMEM, fetal bovine serum, and Trizol™ were obtained from Thermo Fisher Scientific (Waltham, MA, USA). Trypsin-EDTA (0.25%), phosphate-buffered saline (PBS), and gentamicin/colistin double antiserum were purchased from HyClone (Logan, UT, USA). Cell cryopreservation solution was obtained from Promega (Madison, WI, USA). The bovine α-casein ELISA kit was purchased from Shanghai Jianglai Biotechnology Co., Ltd. (Shanghai, China). DEPC-treated water was obtained from Beyotime Biotechnology (Shanghai, China). The PrimeScript™ RT Reagent Kit (Perfect Real Time) and TB Green^®^ Premix Ex Taq™ (Tli RNaseH Plus) were purchased from Takara Bio (Beijing, China). RIPA total protein lysis buffer, 1% phosphatase inhibitor, BCA protein concentration assay kit, primary antibody diluent, secondary antibody diluent, and SDS-PAGE gel preparation kit were purchased from ASPEN (Wuhan, China).

### 2.2. MAC-T Cell Line

The bovine mammary epithelial cell line (MAC-T) was purchased from Qingqi (Shanghai, China) Biotechnology Development Co., Ltd. This cell line was established by stable transfection of primary bovine mammary alveolar epithelial cells with the SV40 large T-antigen gene. These cells grow as an adherent monolayer and exhibit a typical epithelial-like, cobblestone morphology. All experiments in this study were performed with MAC-T cells at passage 3 (P3) after thawing. To ensure the reliability of the experimental results, routine quality control was performed: mycoplasma testing was negative (as determined by PCR method); cell identity was verified by morphological observation and growth characteristics. Following immortalization, they possess stable proliferative activity and can be cultured for more than 30 generations while maintaining consistent cell morphology and casein synthesis function, making them suitable for studies on lactation regulation and associated signaling pathways.

### 2.3. Experimental Design

This study employed a single-factor design. The synthesis levels of α-casein in MAC-T cells treated with various concentrations of glycine, asparagine, and phenylalanine were measured using ELISA to determine the appropriate concentration range for each amino acid. The specific concentrations of amino acids added to the culture medium for each treatment group are presented in Table 1. Based on these ranges, a central composite design (CCD) of response surface methodology, implemented in Design-Expert software (version 13), was used to generate 20 different amino acid combinations. The optimal culture time for MAC-T cell proliferation was determined using the MTT assay. Third-generation MAC-T cells were cultured for 24 h, followed by 12 h of starvation treatment. Subsequently, the 20 amino acid combinations were added, and samples were collected at the optimal culture time point. The α-casein synthesis levels in each group were measured by ELISA, and the optimal amino acid combination (designated as the MIX group) was identified through response surface analysis. Furthermore, RT-qPCR was performed to assess the effects of the MIX group on α-casein-encoding genes and the PI3K-AKT-mTOR signaling pathway.

To verify the critical role of the PI3K-AKT-mTOR signaling pathway, the specific PI3K inhibitor LY294002 was used in a blocking experiment. The experiment comprised four core treatment groups, a negative control group (0×), the MIX group, the LY294002 group, and the LY294002 + MIX group, with three replicates per group. The specific concentrations of amino acids and LY294002 added to the culture medium for each treatment group are detailed in Table 2. The effects of these treatments on α-casein synthesis, the expression of α-casein-encoding genes, and indicators related to the PI3K-AKT-mTOR signaling pathway were assessed using RT-qPCR and Western blotting.

### 2.4. Response Surface Design Experiment

To determine the optimal combination of glycine, asparagine, and phenylalanine for promoting α-casein synthesis, a three-factor, five-level central composite design (CCD) was implemented using Design-Expert software (version 13). The three independent variables (denoted as A, B, and C) were the concentrations of glycine, asparagine, and phenylalanine, respectively. Based on the appropriate concentration ranges for these three amino acids determined from the single-factor experiment (Gly: 2.209–17.673 mmol/L; Asn: 1.964–15.710 mmol/L; Phe: 0.982–7.855 mmol/L), five levels were selected for each independent variable, coded as −2, −1, 0, +1, and +2 (representing low, intermediate, and high levels). The experimental factors and their corresponding level values are presented in Table 3. The 20 combinations of glycine, asparagine, and phenylalanine generated by the CCD are listed in Table 4.

### 2.5. MTT

The MTT assay was performed to evaluate cell proliferation. Six 96-well plates were prepared corresponding to six time points (2, 4, 6, 12, 24, and 48 h). MAC-T cells were seeded into 96-well plates at a density of 5 × 10^3^ cells per well and cultured in a humidified incubator at 37 °C with 5% CO_2_ for 24 h to allow cell attachment. The culture medium was then discarded, and the cells were subjected to serum-free starvation for 12 h. Subsequently, the starvation medium was removed, and 100 μL of culture medium containing different amino acid combinations (according to the experimental design) was added to each well. Cells were incubated for 2, 4, 6, 12, 24, or 48 h. After each designated incubation period, the culture medium was discarded, and 20 μL of MTT solution (5 mg/mL) was added to each well, followed by incubation at 37 °C for 4 h. The supernatant was then carefully aspirated, and 150 μL of dimethyl sulfoxide (DMSO) was added to each well. The plates were shaken for 10 min to dissolve the formazan crystals. The absorbance (optical density, OD) of each well was measured at 490 nm using an automatic microplate reader. The cell proliferation rate, expressed as the relative growth rate (RGR), was calculated using the following formula:

RGR (%) = (OD_490_ of treatment group/OD_490_ of control group) × 100%

### 2.6. ELISA

The concentration of α-casein, both intracellular and secreted, was measured using an ELISA kit. MAC-T cells were seeded into T25 culture flasks at a density of 6 × 10^6^ cells per flask and cultured for 24 h. The medium was then discarded, and the cells were subjected to serum-free starvation for 12 h. After removal of the starvation medium, cells were treated with various concentrations of glycine, asparagine, and phenylalanine, either individually or in different combination ratios, according to the experimental design. At the optimal time point determined by the MTT assay, the cell culture supernatant was collected to measure the secreted α-casein concentration. The cells were then harvested, resuspended in ice-cold phosphate-buffered saline (PBS), and lysed using an ultrasonic cell disruptor at 80 kHz for 5 min to obtain the intracellular α-casein fraction. The α-casein concentration in both the culture supernatant and the cell lysates was determined using a commercial bovine α-casein ELISA kit, following the manufacturer’s instructions.

### 2.7. RT-qPCR

Total RNA was extracted from cells using TRIzol™ reagent (Thermo Fisher Scientific) according to the standard protocol. In brief, lysates were sequentially treated with chloroform, isopropanol precipitation, and 75% ethanol washes. The resulting RNA pellet was dissolved in DEPC-treated water, and its concentration was measured via spectrophotometry (A_260_).

Reverse transcription was performed using the PrimeScript™ RT Kit (RR037A, Takara, Shiga, Japan) to synthesize cDNA. All primers were designed to span exon-exon junctions to effectively distinguish cDNA from genomic DNA amplification. RNA samples were treated with DNase I (Thermo Fisher Scientific, Waltham, MA, USA) prior to reverse transcription to remove residual genomic DNA. Subsequent qPCR was carried out on a CFX Connect Real-Time System (Bio-Rad, 17005940, Hercules, CA, USA) using TB Green^®^ Premix Ex Taq™ (RR420A, Takara, Shiga, Japan). The thermal cycling program included an initial denaturation at 95 °C for 30 s, followed by 40 cycles of 95 °C for 5 s, 55 °C for 30 s, and 72 °C for 30 s, with a final melt curve analysis. Melting curve analysis was performed after each run (65 °C to 95 °C, with fluorescence acquisition every 0.5 °C) to verify amplicon specificity. All primers yielded single melting peaks with no evidence of non-specific amplification. The specificity of the qPCR products was verified by melting curve analysis (Supplementary Figure S1). β-Actin served as the internal control, and relative gene expression was calculated using the 2^−ΔΔCt^ method. Primer sequences are provided in Table 5.

### 2.8. Western Blot

Cells were washed twice with ice-cold PBS, and 100 μL of RIPA lysis buffer (containing 1% PMSF and 1% phosphatase inhibitor cocktail) was added to each well of a 6-well plate. After lysis for 3–5 min, cells were scraped, transferred to microcentrifuge tubes, and incubated on ice for 30 min with vortexing every 10 min. The lysates were then centrifuged at 12,000× *g* for 5 min at 4 °C, and the supernatant was collected as total protein. Protein concentrations were determined using a BCA kit, and samples were normalized to equal concentrations. Equal amounts of protein (40 μg per lane) were separated by 10% SDS-PAGE (80 V for 40 min in the stacking gel, followed by 120 V for 100 min in the resolving gel). The separated proteins were then wet-transferred onto a PVDF membrane (activated with methanol for 15 s) under constant current (300 mA) for 90 min in an ice bath. The membrane was blocked with 5% non-fat dry milk in TBST for 1 h at room temperature, followed by overnight incubation at 4 °C with the primary antibody. After three washes with TBST, the membrane was incubated with HRP-conjugated secondary antibody for 30 min at room temperature, followed by four washes with TBST. Protein bands were visualized using an ECL chemiluminescence substrate (A:B = 1:1) and imaged. The optical density of each band was analyzed using AlphaEaseFC software 4.0.0. Antibody information is shown in Table 6.

### 2.9. Statistical Analysis

All data were processed using Microsoft Excel 2016. Statistical analyses were performed with SPSS 19.0 using a one-way ANOVA, and post hoc comparisons among means were carried out with Duncan’s multiple range test. GraphPad Prism 9.5 was used for graphical visualization. Statistical significance was set at *p* < 0.05, and highly significant differences at *p* < 0.01. All data are shown as mean ± standard error of the mean (SEM).

## 3. Results

### 3.1. α-Casein Synthesis and Secretion in MAC-T in Response to Single Amino Acids

**Figure 1:**
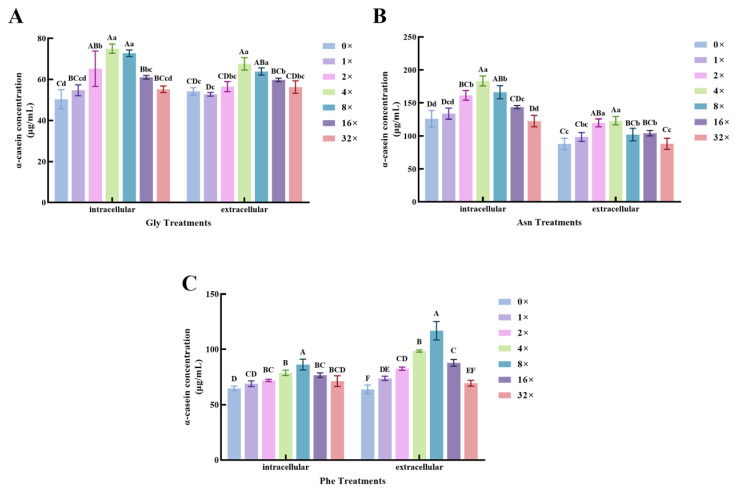
Dose-dependent Effects of Glycine, Asparagine, and Phenylalanine on α-Casein Synthesis and Secretion in MAC-T. MAC-T were treated with different concentrations (0×, 1×, 2×, 4×, 8×, 16×, and 32×) of each amino acid. (**A**) Effects of glycine; (**B**) effects of asparagine; (**C**) effects of phenylalanine. For each panel, the intracellular α-casein synthesis level (synthesis) and the α-casein concentration in the culture supernatant (secretion) were measured by ELISA. Data are presented as mean ± SEM (*n* = 3). Different uppercase letters indicate highly significant differences among groups (*p* < 0.01), and different lowercase letters indicate significant differences among groups (*p* < 0.05).

With increasing concentrations of glycine, asparagine, and phenylalanine, both the synthesis and secretion of α-casein exhibited a biphasic dose–response pattern, characterized by an initial increase followed by a decrease. Compared with the 0× control group, the addition of glycine at 2×, 4×, 8×, and 16× significantly upregulated α-casein synthesis (*p* < 0.01, Figure 1A). The 4× glycine group achieved the highest synthesis level, which was significantly higher than that of the 2× and 16× groups (*p* < 0.05, Figure 1A) and 3.06% higher than that of the 8× group. Regarding α-casein secretion, the 4× and 8× glycine groups showed a highly significant increase (*p* < 0.01, Figure 1A), and the 16× group showed a significant increase (*p* < 0.05, Figure 1A). The 4× glycine group exhibited the highest secretion level, significantly surpassing all other groups (*p* < 0.05).For asparagine, supplementation at 2×, 4×, and 8× resulted in a highly significant upregulation of α-casein synthesis (*p* < 0.01, Figure 1B), and the 16× group also showed a significant increase (*p* < 0.05, Figure 1B). The 2× and 4× asparagine groups showed a highly significant increase in α-casein secretion (*p* < 0.01, Figure 1B), while the 8× and 16× groups showed a significant increase (*p* < 0.05, Figure 1B). The 4× asparagine group achieved the highest levels of both synthesis and secretion, significantly higher than all other groups (*p* < 0.05, Figure 1B).Regarding phenylalanine, supplementation at 2×, 4×, 8×, and 16× significantly upregulated α-casein synthesis (*p* < 0.01, Figure 1C). For secretion, all tested concentrations from 1× to 16× resulted in highly significant upregulation (*p* < 0.01, Figure 1C). The 8× phenylalanine group showed the highest levels of both synthesis and secretion, which were significantly higher than those of all other groups (*p* < 0.01, Figure 1C). Based on these results, the appropriate concentration ranges for promoting α-casein synthesis in MAC-T cells were determined as 2× to 16× for glycine, 2× to 16× for asparagine, and 2× to 16× for phenylalanine, with the optimal supplementation levels being 4× for glycine, 4× for asparagine, and 8× for phenylalanine.

### 3.2. Relative Proliferation Rate of MAC-T in Response to Different Combinations of Glycine, Asparagine, and Phenylalanine

**Figure 2:**
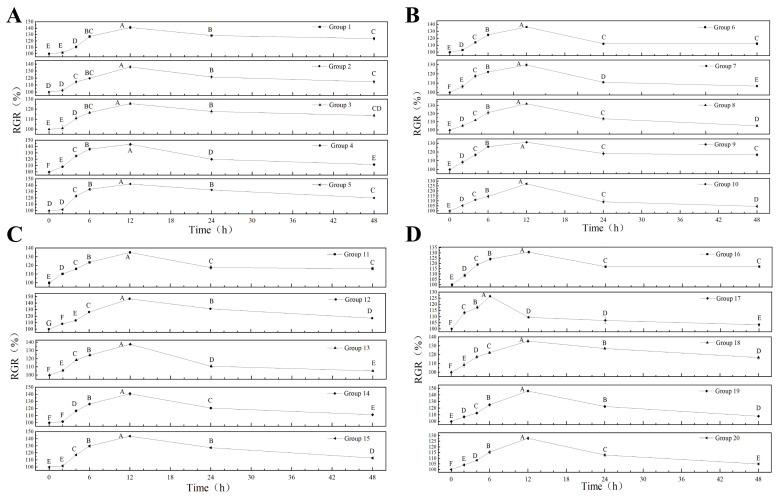
Relative Proliferation Curves of MAC-T Treated with Different Combinations of Glycine, Asparagine, and Phenylalanine. (**A**) Groups 1–5; (**B**) groups 6–10; (**C**) groups 11–15; (**D**) groups 16–20. MAC-T were treated with 20 different amino acid combinations (groups 1–20) according to the central composite design. Cell proliferation was assessed by MTT assay at 2, 4, 6, 12, 24, and 48 h. The relative proliferation rate was calculated as (OD_490_ of treatment group/OD_490_ of control group) × 100%. Data are presented as mean ± SEM (*n* = 3). Different uppercase letters indicate highly significant differences among time points within each group (*p* < 0.01).

As shown in Figure 2, when MAC-T cells were treated with different combinations of glycine, asparagine, and phenylalanine at various ratios, the relative proliferation rate initially increased and then decreased with prolonged culture time. Compared with 0 h, treatment groups 1, 2, 3, 5, 14, and 15 exhibited a highly significant increase in relative proliferation rate between 4 and 48 h (*p* < 0.01). Treatment groups 4, 6, 7, 8, 9, 10, 11, 12, 13, 16, 17, 18, 19, and 20 showed a highly significant increase between 2 and 48 h (*p* < 0.01). For all treatment groups except group 17, the relative proliferation rate peaked at 12 h, with a value that was significantly higher than at other time points (*p* < 0.01). For group 17, the relative proliferation rate peaked at 6 h, with a value that was significantly higher than at other time points (*p* < 0.01). Based on these results, 12 h was selected as the sample collection time for subsequent experiments.

### 3.3. Screening of the Optimal Combination of Glycine, Asparagine, and Phenylalanine for α-Casein Synthesis Using Response Surface Design

To investigate the effects of glycine, asparagine, and phenylalanine on α-casein synthesis in MAC-T cells, different concentrations and ratios of these three amino acids were added to the culture medium. According to the response surface methodology optimization, the highest α-casein synthesis level was achieved at a glycine concentration of 9.895 mmol/L, an asparagine concentration of 7.018 mmol/L, and a phenylalanine concentration of 5.865 mmol/L, corresponding to a molar ratio of 1.69:1.20:1.00 (glycine:asparagine:phenylalanine).

As shown in Table 7 and Table 8, different concentrations and ratios of glycine, asparagine, and phenylalanine were added to the culture medium of MAC-T cells. As shown in Table 9, the response surface regression model revealed that these three amino acids significantly affected α-casein synthesis (*p* < 0.01, R^2^ = 0.9611). The lack-of-fit term was not significant (*p* = 0.6336 > 0.05), indicating that the model fit was good and that unknown factors had minimal interference with the experimental results. Therefore, this model can be used to analyze and predict α-casein synthesis levels. From the analysis of variance for each factor, the first-order terms A (glycine), B (asparagine), and C (phenylalanine) all exhibited a highly significant effect on α-casein synthesis (*p* < 0.01). Among them, phenylalanine had the largest F-value (F = 23.27, *p* = 0.0007), indicating that it exerted the most prominent effect on α-casein synthesis, followed by glycine (F = 15.82, *p* = 0.0026) and asparagine (F = 12.39, *p* = 0.0055). Using Design-Expert 13 software, multiple regression fitting was performed on the α-casein synthesis levels from the 20 amino acid combinations. The quadratic polynomial regression equation relating glycine (A), asparagine (B), and phenylalanine (C) to α-casein synthesis (Y) is:

Y = 143.2 + 1.98 × A + 6.50 × B + 7.84 × C + 0.17 × AB + 0.65 × AC − 0.49×BC − 0.35×A^2^ − 0.38×B^2^ − 0.92×C^2^

**Figure 3:**
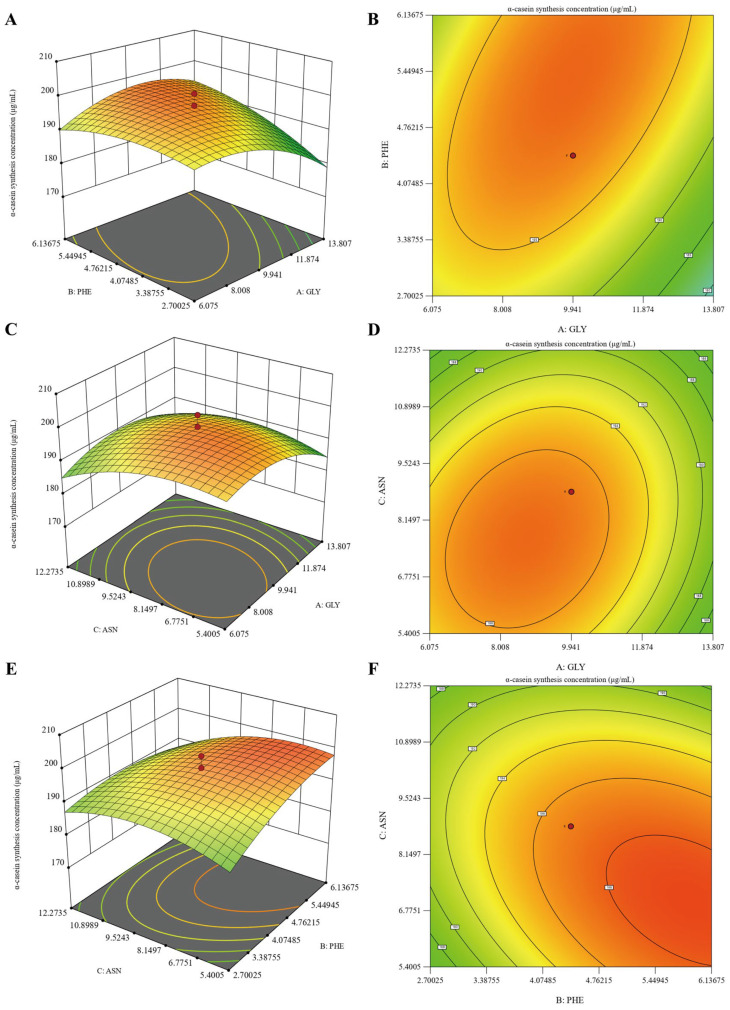
Response Surface and Contour Plots Showing the Interactive Effects of Glycine, Asparagine, and Phenylalanine on α-Casein Synthesis in MAC-T. (**A**,**B**) Interactive effects of glycine and asparagine on α-casein synthesis. (**C**,**D**) Interactive effects of glycine and phenylalanine on α-casein synthesis. (**E**,**F**) Interactive effects of asparagine and phenylalanine on α-casein synthesis. The left panels (**A**,**C**,**E**) are three-dimensional response surface plots, and the right panels (**B**,**D**,**F**) are corresponding contour plots.

As shown in Figure 3A,B, glycine (A) and asparagine (B) exhibited a significant interactive effect on α-casein synthesis (*p* < 0.05). The response surface plot revealed a clear peak, where α-casein synthesis initially increased with increasing concentrations of both factors, followed by a decline, indicating optimal concentration ranges for each amino acid. At low glycine levels, increasing asparagine concentration had a relatively moderate effect on α-casein synthesis. However, at high glycine levels, increasing asparagine caused a sharp decline in synthesis, suggesting that excessive glycine amplifies the inhibitory effect of asparagine. The distinct elliptical shape of the contour plot confirmed the significance of the interaction (*p* < 0.05). Denser contour lines along the A-axis (glycine) compared to the B-axis (asparagine) indicated that glycine concentration had a greater impact on α-casein synthesis within the tested range. A relatively high level of α-casein synthesis was achieved with glycine at 9.5–10.5 mmol/L and asparagine at 6.8–7.3 mmol/L.

As shown in Figure 3C,D, glycine (A) and phenylalanine (C) exhibited a highly significant interactive effect on α-casein synthesis (*p* < 0.01). The response surface plot revealed a clear peak, where α-casein synthesis initially increased with increasing concentrations of both factors, followed by a decline, indicating optimal concentration ranges for each amino acid. At low glycine levels, increasing phenylalanine concentration had a relatively moderate effect on α-casein synthesis. At medium glycine levels, increasing phenylalanine significantly enhanced synthesis. However, at high glycine levels, the promoting effect of phenylalanine was attenuated. The contour plot exhibited a highly elongated elliptical shape, almost parallel to the diagonal, further supporting the highly significant interaction (*p* < 0.001). Denser contour lines along the C-axis (phenylalanine) compared to the A-axis (glycine) indicated that phenylalanine concentration had a greater impact on α-casein synthesis within the tested range. A relatively high level of α-casein synthesis was achieved with glycine at 9.5–10.5 mmol/L and phenylalanine at 5.5–6.0 mmol/L.

As shown in Figure 3E,F, asparagine (B) and phenylalanine (C) exhibited a highly significant interactive effect on α-casein synthesis (*p* < 0.01). The response surface plot revealed a clear peak, where α-casein synthesis initially increased with increasing concentrations of both factors, followed by a decline, indicating optimal concentration ranges for each amino acid. At low phenylalanine levels, increasing asparagine concentration resulted in a gradual increase followed by a gradual decrease in α-casein synthesis. At high phenylalanine levels, increasing asparagine concentration led to a sharp increase followed by a sharp decline. The contour plot exhibited a distinct elliptical shape, confirming the highly significant interaction (*p* < 0.01). Denser contour lines along the C-axis (phenylalanine) compared to the B-axis (asparagine) indicated that phenylalanine concentration had a greater impact on α-casein synthesis within the tested range. A relatively high level of α-casein synthesis was achieved with asparagine at 6.8–7.3 mmol/L and phenylalanine at 5.5–6.0 mmol/L.

### 3.4. α-Casein Synthesis and Secretion in MAC-T in Response to Optimal Levels and Ratios of Glycine, Asparagine, and Phenylalanine

**Figure 4:**
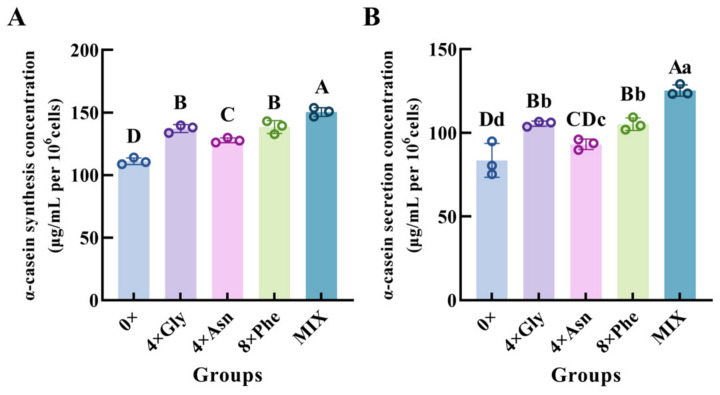
α-Casein Synthesis and Secretion in MAC-T Cells in Response to Optimal Levels of Glycine, Asparagine, and Phenylalanine and Their Optimal Combination (MIX). MAC-T cells were treated with 4× Gly, 4× Asn, 8× Phe, or the MIX combination (glycine: 9.898 mmol/L, asparagine: 7.014 mmol/L, phenylalanine: 5.865 mmol/L). The 0× group served as the negative control. (**A**) Intracellular α-casein synthesis, (**B**) α-casein secretion in the culture supernatant. Both were measured by ELISA. α-Casein concentrations measured by ELISA were normalized to viable cell number and expressed as μg/mL per 10^6^ cells. Data are presented as mean ± SEM (*n* = 3). Different uppercase letters indicate highly significant differences among groups (*p* < 0.01), and different lowercase letters indicate significant differences among groups (*p* < 0.05).

As shown in Figure 4, compared with the 0× control group, the 4× Gly, 4× Asn, 8× Phe, and MIX groups all exhibited highly significant increases in α-casein synthesis (*p* < 0.01). Compared with the 0× control group, α-casein secretion was highly significantly increased in the 4× Gly, 8× Phe, and MIX groups (*p* < 0.01), and significantly increased in the 4× Asn group (*p* < 0.05). Furthermore, the MIX group showed significantly higher levels of both synthesis and secretion than the 4× Gly, 4× Asn, and 8× Phe groups (*p* < 0.01).

### 3.5. α-Casein-Encoding Gene Expression in MAC-T in Response to the Optimal Amino Acid Combination

**Figure 5:**
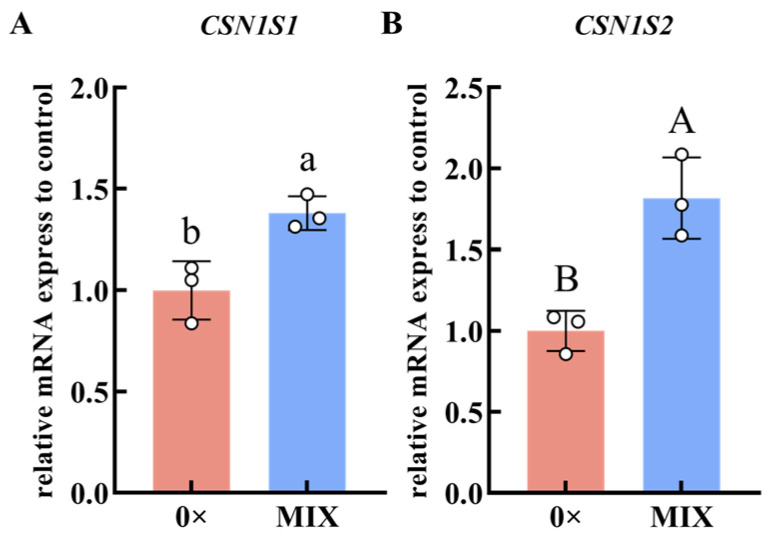
α-Casein-encoding Gene Expression in MAC-T in Response to the Optimal Amino Acid Combination (MIX). MAC-T cells were treated with the MIX combination. The 0× group served as the negative control. The relative expression levels of CSN1S1 and CSN1S2 were measured by RT-qPCR and normalized to β-actin. (**A**) CSN1S1 expression, (**B**) CSN1S2 expression. Data are presented as mean ± SEM (*n* = 3). Different uppercase letters indicate highly significant differences among groups (*p* < 0.01), and different lowercase letters indicate significant differences among groups (*p* < 0.05).

As shown in Figure 5, compared with the 0× control group, the MIX group exhibited a significantly increased relative expression level of the *CSN1S1* gene (*p* < 0.05) and a highly significantly increased relative expression level of the *CSN1S2* gene (*p* < 0.01).

### 3.6. PI3K-AKT-mTOR Signaling Pathway Gene Expression in MAC-T in Response to the Optimal Amino Acid Combination

**Figure 6:**
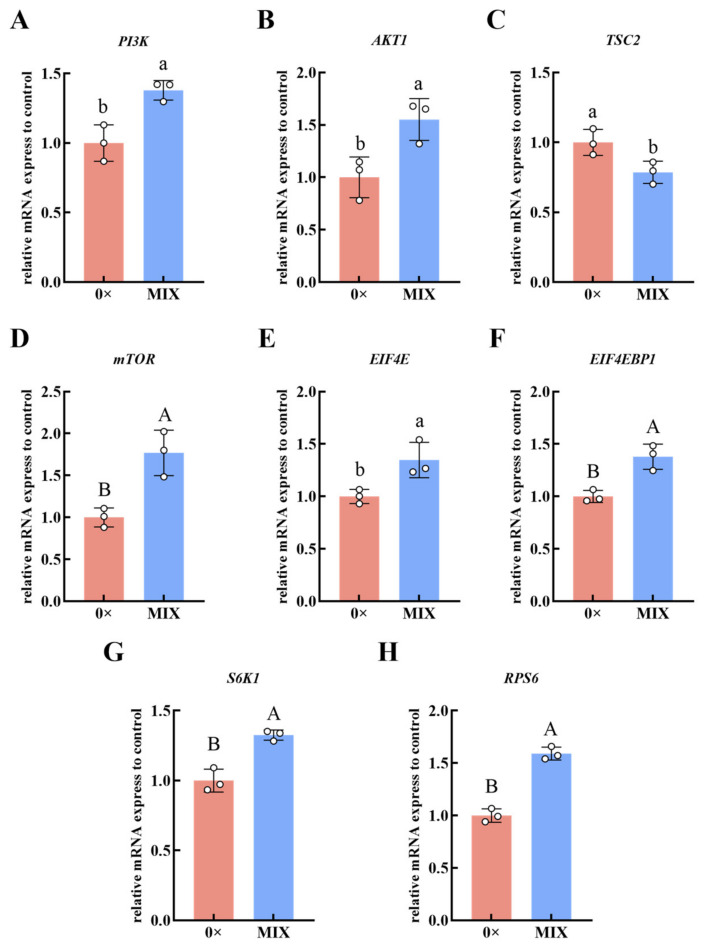
PI3K-AKT-mTOR signaling pathway gene expression in MAC-T cells in response to the optimal amino acid combination (MIX). The 0× group served as the negative control. The relative expression levels of the following genes were measured by RT-qPCR and normalized to β-actin: (**A**) *PI3K*; (**B**) *AKT1*; (**C**) *TSC2*; (**D**) *mTOR*; (**E**) *EIF4EBP1*; (**F**) *EIF4E*; (**G**) *S6K1*; (**H**) *RPS6*. Data are presented as mean ± SEM (*n* = 3). Different uppercase letters indicate highly significant differences among groups (*p* < 0.01), and different lowercase letters indicate significant differences among groups (*p* < 0.05).

As shown in Figure 6, compared with the 0× control group, the MIX group exhibited highly significant increases in the relative expression levels of *mTOR*, *EIF4EBP1*, *S6K1*, and *RPS6* genes (*p* < 0.01), significant increases in *PI3K*, *AKT1*, and *EIF4E* genes (*p* < 0.05), and a significant decrease in the *TSC2* gene (*p* < 0.05).

### 3.7. Inhibition of PI3K to Verify Its Role in Mediating α-Casein Synthesis in MAC-T in Response to the Optimal Amino Acid Combination

#### 3.7.1. Inhibition of PI3K to Verify Its Role in Mediating α-Casein-Encoding Gene Expression in MAC-T in Response to the Optimal Amino Acid Combination

**Figure 7:**
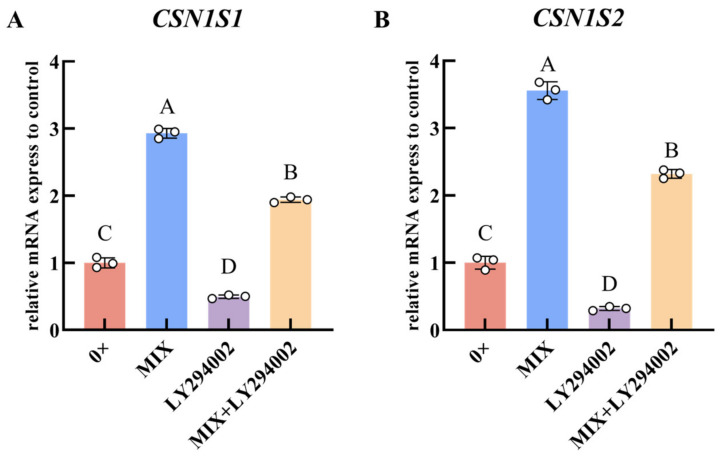
Inhibition of PI3K Blocks α-Casein-encoding Gene Expression Induced by the Optimal Amino Acid Combination. MAC-T cells were treated with the MIX combination in the presence or absence of LY294002 (50 μmol/L). The 0× group served as the negative control. The relative expression levels of α-casein genes were measured by RT-qPCR and normalized to β-actin. (**A**) *CSN1S1* expression; (**B**) *CSN1S2* expression. Data are presented as mean ± SEM (*n* = 3). Different uppercase letters indicate highly significant differences among groups (*p* < 0.01).

As shown in Figure 7, compared with the 0× control group, the MIX group exhibited highly significant increases in the relative expression levels of *CSN1S1* and *CSN1S2* genes (*p* < 0.01). Furthermore, the addition of LY294002 (a specific PI3K inhibitor) to the medium containing the optimal amino acid combination effectively inhibited this activating effect. Compared with the MIX group, the MIX + LY294002 group showed highly significant downregulation of *CSN1S1* and *CSN1S2* gene expression (*p* < 0.01).

#### 3.7.2. Inhibition of PI3K to Verify Its Role in Mediating Signaling Pathway Gene Expression in MAC-T in Response to the Optimal Amino Acid Combination

**Figure 8:**
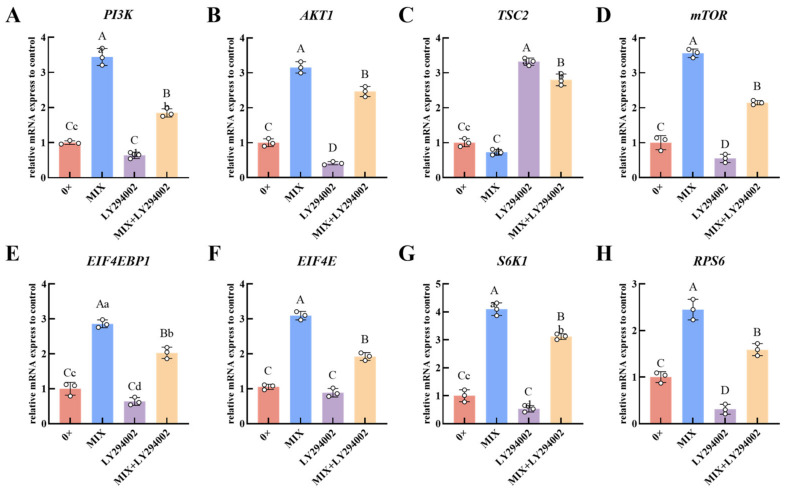
PI3K Inhibition Attenuates PI3K-AKT-mTOR Signaling Pathway Gene Expression in MAC-T in Response to the Optimal Amino Acid Combination. MAC-T were treated with the MIX combination in the presence or absence of LY294002 (50 μmol/L). The 0× group served as the negative control. The relative expression levels of the following genes were measured by RT-qPCR and normalized to β-actin: (**A**) *PI3K*; (**B**) *AKT1*; (**C**) *TSC2*; (**D**) *mTOR*; (**E**) *EIF4EBP1*; (**F**) *EIF4E*; (**G**) *S6K1*; (**H**) *RPS6*. Data are presented as mean ± SEM (*n* = 3). Different uppercase letters indicate highly significant differences among groups (*p* < 0.01), and different lowercase letters indicate significant differences among groups (*p* < 0.05).

As shown in Figure 8, compared with the 0× control group, the MIX group exhibited highly significant increases in the relative expression levels of *PI3K*, *AKT1*, *mTOR*, *EIF4EBP1*, *EIF4E*, *S6K1*, and *RPS6* genes (*p* < 0.01), and a significant decrease in the *TSC2* gene (*p* < 0.05). Furthermore, the addition of LY294002 to the medium containing the optimal amino acid combination effectively inhibited the activating effect of the MIX on the PI3K-AKT-mTOR signaling pathway. Compared with the MIX group, the MIX + LY294002 group showed highly significant decreases in the relative expression levels of *PI3K*, *AKT1*, *mTOR*, *EIF4EBP1*, *EIF4E*, *S6K1*, and *RPS6* genes (*p* < 0.01), and a highly significant increase in the *TSC2* gene (*p* < 0.01).

#### 3.7.3. Inhibition of PI3K to Verify Its Role in Mediating Signaling Pathway Protein Phosphorylation in MAC-T Cells in Response to the Optimal Amino Acid Combination

**Figure 9:**
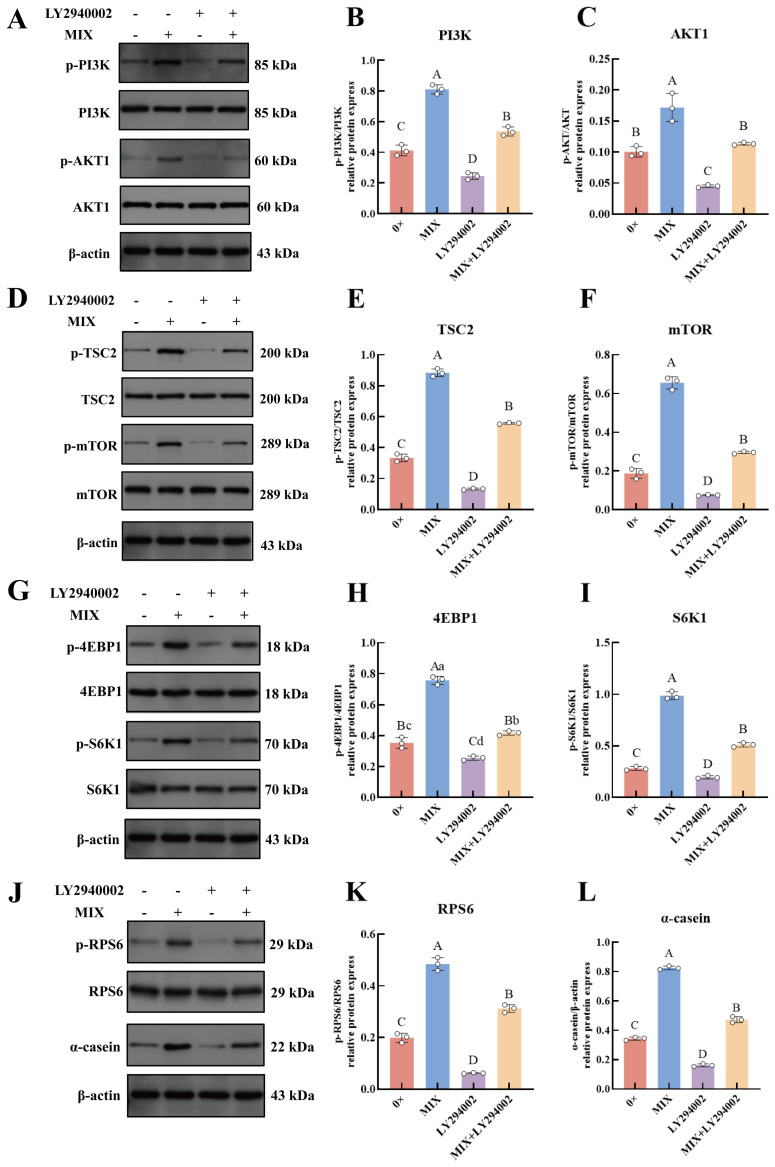
PI3K inhibition attenuates PI3K-AKT-mTOR signaling pathway protein phosphorylation and α-casein expression in MAC-T cells in response to the optimal amino acid combination. MAC-T cells were treated with the MIX combination in the presence or absence of LY294002 (50 μmol/L). The 0× group served as the negative control. (**A**) Western blot bands for PI3K and AKT; (**B**) phosphorylated PI3K (p-PI3K) levels; (**C**) phosphorylated AKT1 (p-AKT1) levels; (**D**) Western blot bands for TSC2 and mTOR; (**E**) phosphorylated TSC2 (p-TSC2) levels; (**F**) phosphorylated mTOR (p-mTOR) levels; (**G**) Western blot bands for 4EBP1 and S6K1; (**H**) phosphorylated 4EBP1 (p-4EBP1) levels; (**I**) phosphorylated S6K1 (p-S6K1) levels; (**J**) Western blot bands for RPS6 and α-casein; (**K**) phosphorylated RPS6 (p-RPS6) levels; (**L**) α-casein total protein expression. All protein levels were measured by Western blotting and normalized to β-actin. Data are presented as mean ± SEM (n = 3). Different uppercase letters indicate highly significant differences among groups (*p* < 0.01), and different lowercase letters indicate significant differences among groups (*p* < 0.05).

As shown in Figure 9, compared with the 0× control group, the MIX group exhibited highly significant increases in the phosphorylation levels of PI3K, AKT, TSC2, mTOR, 4EBP1, S6K1, and RPS6 proteins, as well as the expression level of α-casein protein (*p* < 0.01). Furthermore, the addition of LY294002 to the medium containing the optimal amino acid combination effectively inhibited the activating effect of the MIX on the PI3K-AKT-mTOR signaling pathway. Compared with the MIX group, the MIX + LY294002 group showed highly significant decreases in the phosphorylation levels of PI3K, AKT, TSC2, mTOR, 4EBP1, S6K1, and RPS6 proteins, as well as the expression level of α-casein protein (*p* < 0.01).

## 4. Discussion

In this study, the regulation of α-casein synthesis and secretion by glycine, asparagine, and phenylalanine exhibited a typical biphasic dose–response pattern, which is consistent with previous findings on various amino acids in mammary epithelial cells [10,11,12]. The optimal concentrations differed among the three amino acids: glycine and asparagine reached their peaks at the 4× level, whereas phenylalanine peaked at the 8× level. The observation that casein synthesis and secretion did not continuously increase with increasing concentrations of individual amino acids can be explained by the fact that excessive amounts of a single amino acid disrupt the intracellular amino acid pool balance and competitively inhibit the transport and utilization of other essential amino acids (such as lysine and methionine), thereby limiting casein synthesis [13]. Thus, the promoting effect of a single amino acid has an upper concentration limit, and simply increasing the supply of one amino acid is insufficient to continuously enhance casein synthesis efficiency. This limitation suggests that efficient milk protein synthesis may depend more on the synergistic supply of multiple amino acids rather than the isolated effect of any single amino acid. In vivo, amino acids do not exist in isolation but function within a complex network of multiple amino acids, where synergistic and antagonistic interactions make the overall effect of an amino acid combination far exceed the simple addition of individual effects. Gao et al. [14] demonstrated that a combination of histidine, lysine, methionine, and leucine synergistically promotes β-casein synthesis in bovine mammary epithelial cells. The results of the present study showed that, compared with the 0× control group, the MIX group exhibited highly significant increases in both α-casein synthesis and secretion. Moreover, the MIX group showed significantly higher levels of both synthesis and secretion than the 4× Gly, 4× Asn, and 8× Phe groups. These findings indicate that the optimal ratio of the three amino acids exerts a significant synergistic effect on milk protein synthesis and secretion, outperforming the optimal concentration of any single amino acid.

The synergistic effect of the MIX group observed in this study may arise from the combined action of multiple mechanisms. The optimal ratio of the three amino acids may synergistically upregulate the expression of amino acid transporters such as *LAT1* and *ASCT2* [15], thereby enhancing amino acid uptake efficiency and providing sufficient substrate support for intracellular protein synthesis. Additionally, glycine, as a key participant in one-carbon unit metabolism and glutathione synthesis, may provide auxiliary support for protein synthesis and help maintain cellular redox balance [16]. Asparagine plays an important role in maintaining cell proliferation and metabolic homeostasis [17], while phenylalanine participates in the regulation of milk protein synthesis by modulating amino acid transport and the mTOR signaling pathway [18]. The complementary functions of these three amino acids collectively enhance α-casein synthesis efficiency and promote its secretion into the extracellular space.

The transcriptional efficiency of casein-encoding genes is a key molecular determinant of milk protein synthesis potential. In mammary epithelial cells, amino acids not only serve as substrates for protein synthesis but also regulate the transcriptional activity of casein genes. Studies have shown that the regulatory effect of amino acid combinations on casein gene expression often exceeds the simple additive effect of individual amino acids, and this non-additive nature is an important manifestation of synergy among amino acids. Wang et al. [19] found that when lysine and methionine were mixed at a ratio of 3:1, the expression of multiple casein genes, including *CSN1S1* and *CSN1S2*, was significantly upregulated, indicating that an appropriate amino acid combination can synergistically activate casein synthesis at the transcriptional level. Yang et al. [20] further confirmed that an optimized amino acid combination promotes the expression of α-casein-encoding genes, with a greater upregulation effect than that achieved by single amino acids. Xu et al. [21] also demonstrated, through multi-omics analysis, that different essential amino acids exert distinct effects on the transcriptome of bovine mammary epithelial cells (BMECs). Collectively, these findings suggest that optimizing amino acid combination ratios is crucial for maximizing milk protein synthesis efficiency, and that a well-designed combination may achieve more effective overall activation through synergistic effects. The results of the present study showed that the MIX group significantly increased the expression levels of α-casein-encoding genes, indicating that the optimal ratio of glycine, asparagine, and phenylalanine synergistically activates casein gene expression at the transcriptional level. This observation is highly consistent with the protein-level results described above. Notably, the upregulation of *CSN1S2* by the MIX group was stronger than that of *CSN1S1*, suggesting that the two casein subtypes exhibit differential sensitivity to amino acid combinations. This phenomenon may be attributed to differences in the regulatory elements within the promoter regions of *CSN1S1* and *CSN1S2*. Specifically, the binding sites and affinities for transcription factors such as STAT5 and C/EBPβ differ between the two gene promoters, which may lead to distinct response patterns to nutritional signals [22]. The significant upregulation of both *CSN1S1* and *CSN1S2* expression by the MIX group in this study strongly demonstrates the central role of specific amino acid combinations in activating casein gene transcription, thereby providing an important molecular basis for understanding how optimally formulated amino acid combinations promote milk protein synthesis.

The PI3K-AKT-mTOR pathway serves as a core hub for integrating amino acid and other nutrient signals and regulating protein synthesis within cells [23]. Within this pathway, PI3K and AKT play positive regulatory roles, whereas TSC2 functions as a negative regulator by inhibiting Rheb activity and thereby blocking mTOR activation [24]. In the present study, the optimal combination of glycine, asparagine, and phenylalanine significantly upregulated the expression of *PI3K* and *AKT1* genes in MAC-T cells while significantly downregulating *TSC2* gene expression, indicating that this amino acid combination can synergistically enhance positive signals and relieve negative inhibition at the transcriptional level, thereby creating favorable conditions for downstream mTOR activation. Han et al. [25] confirmed that amino acids such as methionine and leucine can regulate mTOR gene expression in mammary epithelial cells by activating the PI3K signaling pathway, which is consistent with the findings of the present study. The downregulation of *TSC2* gene expression plays a key role in pathway activation. TSC2 protein is a core negative regulatory node for mTORC1 activation [26], and its reduced expression directly weakens the inhibition of Rheb by the TSC1/2 complex, laying the foundation for downstream mTOR activation.

Downstream of the pathway, mTOR initiates protein translation by regulating effectors such as S6K1, 4EBP1, and eIF4E [27,28]. In the present study, treatment with the optimal amino acid ratio significantly upregulated the gene expression of *mTOR*, *EIF4EBP1*, *S6K1*, and *RPS6*, and also significantly increased *EIF4E* expression, indicating that this amino acid combination can transmit the activation signal in a cascade manner to downstream targets, thereby promoting protein synthesis at the transcriptional level. Burgos et al. [29] reported that amino acid combinations upregulate the gene expression of mTORC1 and its downstream targets to promote milk protein synthesis, which is consistent with the findings of the present study. Notably, the activation levels of different downstream effectors varied. The expression levels of *mTOR*, *EIF4EBP1*, *S6K1*, and *RPS6* were highly significantly increased, whereas *EIF4E* expression was only significantly upregulated. This difference is attributable to the distinct functional roles of each factor in translation regulation. As a core component of the translation initiation complex, eIF4E activity is primarily regulated by post-translational modifications, such as its binding to and dissociation from 4EBP1, rather than by transcriptional regulation [30,31]. Therefore, the highly significant upregulation of *EIF4EBP1* expression by the MIX group provides a molecular basis for the subsequent phosphorylation of 4EBP1, which in turn releases eIF4E and initiates the assembly of the translation initiation complex. Dai et al. [32] demonstrated that essential amino acid combinations promote β-casein synthesis by enhancing mTOR signaling. In the present study, the optimal combination of glycine, asparagine, and phenylalanine synergistically activated the upstream PI3K-AKT pathway, amplified and transmitted the signal cascade to downstream effectors, ultimately achieving multi-target regulation of α-casein synthesis.

To verify whether the optimal combination of glycine, asparagine, and phenylalanine promotes α-casein synthesis through the PI3K-AKT-mTOR pathway, the present study employed the PI3K-specific inhibitor LY294002 for blocking validation. LY294002 inhibits the generation of phosphatidylinositol (3,4,5)-trisphosphate (PIP_3_) by competitively binding to the ATP-binding site of the PI3K catalytic subunit, thereby blocking the activation of downstream AKT and mTOR [33]. Following the addition of LY294002 to block the PI3K-AKT-mTOR signaling pathway, the expression levels of *CSN1S1* and *CSN1S2* in the MIX + LY294002 group were significantly lower than those in the MIX group. In bovine mammary epithelial cells, the expression of casein genes is regulated by multiple transcription factors, and the activity of these transcription factors can be indirectly modulated by the PI3K-AKT pathway [34]. Moreover, LY294002 has been shown to inhibit the expression of multiple casein-encoding genes by blocking the PI3K pathway [35,36,37]. Collectively, the downregulation of *CSN1S1* and *CSN1S2* by LY294002 in the present study further demonstrates that the optimal combination of glycine, asparagine, and phenylalanine regulates downstream transcription factors by activating the PI3K-AKT pathway, thereby promoting casein gene transcription.

Compared with the MIX group, the combined treatment with LY294002 and MIX (MIX + LY294002) resulted in highly significant decreases in the relative expression levels of *PI3K*, *AKT1*, *mTOR*, *EIF4EBP1*, *EIF4E*, *S6K1*, and *RPS6* genes, while the relative expression level of *TSC2* was highly significantly increased. These findings are consistent with those of Tian et al. [36], who reported that LY294002-mediated inhibition of the PI3K-AKT-mTOR pathway suppressed the expression of key genes involved in the mTOR pathway, including *AKT*, *Rheb*, *PRAS40*, and *S6K1*. At the protein level, MIX treatment significantly increased the phosphorylation levels of PI3K-AKT-mTOR signaling pathway-related proteins and upregulated α-casein expression. Following the addition of LY294002, the phosphorylation levels of these signaling pathway proteins were highly significantly reduced, accompanied by a highly significant decrease in α-casein synthesis. These results indicate that the optimal amino acid combination activates PI3K, which in turn phosphorylates and activates AKT1. Activated AKT1 then phosphorylates TSC2, leading to its functional inactivation, thereby relieving its inhibition of Rheb and activating mTORC1 along with its downstream effectors 4EBP1 and S6K1, ultimately initiating the translation process [38]. Notably, although all measured parameters in the MIX + LY294002 group were highly significantly lower than those in the MIX group, they remained significantly higher than those in the LY294002-alone treatment group. Amemiya et al. [39] further revealed that amino acids can activate the TSC2-Rheb axis by increasing intracellular Ca^2+^ concentration through calmodulin activation, thereby directly promoting mTORC1 activation. Meng et al. [40] demonstrated that glutamine and asparagine activate mTORC1 via the Arf1 pathway, a process that is independent of Rag GTPase and the PI3K-AKT signaling axis. These observations suggest that the optimal combination of glycine, asparagine, and phenylalanine may promote α-casein synthesis in MAC-T cells not only through the PI3K-AKT-mTOR signaling pathway but also via additional auxiliary pathways or compensatory mechanisms, warranting further investigation.

In vitro cell culture systems inherently lack the complex metabolic regulatory networks present in vivo—including digestion, absorption, hepatic metabolism, renal excretion, hormonal regulation, and transmembrane transport—and therefore typically require higher extracellular amino acid concentrations to effectively activate signaling pathways and induce detectable biological responses within a defined time frame. It is therefore not surprising that the optimal concentrations identified in the present study (9.898 mmol/L glycine, 7.014 mmol/L asparagine, and 5.865 mmol/L phenylalanine) are substantially higher than the physiological plasma concentrations reported for lactating dairy cows (glycine approximately 211–332 μmol/L, phenylalanine approximately 66–88 μmol/L, asparagine approximately 7.9–9.8 μmol/L) [41]. What is biologically meaningful, however, is the optimal molar ratio among these three amino acids (glycine:asparagine:phenylalanine = 1.69:1.20:1.00), rather than the absolute concentration per se. This ratio provides a testable hypothesis for future in vivo studies aimed at optimizing the amino acid profile of diets or rumen-protected formulations to enhance milk protein synthesis. In this context, our findings are best interpreted as a mechanistic proof-of-concept showing that a specific amino acid ratio can synergistically activate the PI3K-AKT-mTOR pathway and promote α-casein synthesis. To the best of our knowledge, this is the first systematic report of such an effect involving glycine, asparagine, and phenylalanine in combination. Future studies in lactating dairy cows are warranted to translate this ratio into physiologically achievable concentrations and to evaluate its practical efficacy and economic feasibility.

## 5. Conclusions

In this study, the optimal ratio of glycine, asparagine, and phenylalanine for promoting α-casein synthesis in MAC-T cells was identified using response surface central composite design as 9.898 mmol/L:7.014 mmol/L:5.865 mmol/L, corresponding to a molar ratio of 1.69:1.20:1.00. This optimal ratio exerted a significant synergistic promoting effect on α-casein synthesis and secretion, outperforming any single amino acid. The optimal combination of glycine, asparagine, and phenylalanine upregulated the expression of *CSN1S1* and *CSN1S2* genes and simultaneously activated both the transcription and phosphorylation of genes associated with the PI3K-AKT-mTOR signaling pathway. Blocking experiments using the PI3K-specific inhibitor LY294002 confirmed that the PI3K-AKT-mTOR signaling pathway is the core mechanism mediating the promoting effect of this optimal amino acid combination on α-casein synthesis in MAC-T cells. Collectively, these findings provide a theoretical basis for the application of functional amino acid combinations in precision nutrition strategies for dairy cows. Future studies are warranted to validate the efficacy of this optimal amino acid combination in lactating dairy cows and to evaluate its economic feasibility.

## Figures and Tables

**Table 1:** Concentrations of Glycine, Asparagine, and Phenylalanine Used in the Single-factor Experiment (mmol/L).

Items	Groups						
	0×	1×	2×	4×	8×	16×	32×
Glycine	0.400	1.105	2.209	4.418	8.836	17.673	35.345
Asparagine	0.000	0.982	1.964	3.927	7.855	15.710	31.418
Phenylalanine	0.400	0.491	0.982	1.964	3.927	7.855	15.710

The concentrations are expressed as mmol/L. The “×” values represent multiples of the basal concentration (1×) for each amino acid. Cells were treated with the indicated concentrations for optimal duration before α-casein synthesis was measured. We used high-glucose DMEM (Thermo Fisher Scientific) as the basal culture medium. According to the manufacturer’s formulation, this medium contains basal levels of glycine (0.400 mmol/L) and phenylalanine (0.400 mmol/L), but does not contain asparagine.

**Table 2:** Concentrations of Amino Acids and LY294002 in the Culture Medium for Each Treatment Group in the Blocking Experiment.

Items	Groups			
	0×	MIX	LY294002	LY294002 + MIX
Glycine (mmol/L)	0.400	9.898	0.400	9.898
Asparagine (mmol/L)	0.000	7.014	0.000	7.014
Phenylalanine (mmol/L)	0.400	5.865	0.400	5.865
LY294002 (μmol/L)	0.000	0.000	50.000	50.000
FBS (%)	0.000	0.000	0.000	0.000

All treatments were performed under serum-free conditions (0% FBS). The concentration of LY294002 (50 μmol/L) was determined based on our previous study [4].

**Table 3:** Coded Levels of Experimental Factors in the Response Surface Central Composite Design for Optimizing α-Casein Synthesis.

Factor	Level				
	−2	−1	0	+1	+2
A-Gly (mmol/L)	2.209	6.075	9.941	13.807	17.673
B-Asn (mmol/L)	1.964	5.4005	8.837	12.2735	15.71
C-Phe (mmol/L)	0.982	2.70025	4.4185	6.13675	7.855

Three independent variables (A, B, C) represent the concentrations of glycine, asparagine, and phenylalanine, respectively. Coded levels (−2, −1, 0, +1, +2) correspond to the actual concentrations shown above.

**Table 4:** Central Composite Design Matrix for Optimizing α-Casein Synthesis with Glycine, Asparagine, and Phenylalanine.

Run	A (Gly)	B (Asn)	C (Phe)
1	−1.000	−1.000	−1.000
2	1.000	−1.000	−1.000
3	−1.000	1.000	−1.000
4	1.000	1.000	−1.000
5	−1.000	−1.000	1.000
6	1.000	−1.000	1.000
7	−1.000	1.000	1.000
8	1.000	1.000	1.000
9	−2.000	0.000	0.000
10	2.000	0.000	0.000
11	0.000	−2.000	0.000
12	0.000	2.000	0.000
13	0.000	0.000	−2.000
14	0.000	0.000	2.000
15	0.000	0.000	0.000
16	0.000	0.000	0.000
17	0.000	0.000	0.000
18	0.000	0.000	0.000

A, B, and C represent the coded levels for glycine, asparagine, and phenylalanine, respectively, as defined in Table 3. Runs 15–18 are center points.

**Table 5:** Primer Sequences Used for RT-qPCR.

Gene	Accession No.	Primer Sequence (5′ → 3′)
*PI3K*	XM_024988896	F:ATGGTGATGATTTACGGCAGGATATG R:TAAGGTAGCATCCGAAGGTCCAG
*AKT1*	XM_024981592	F:ATCATGCAGCACCGATTCTT R:AAATACCTGGTGTCCGTCTCA
*TSC2*	XM_059881502	F:GAGACACATCACCTACTTGGAAGAAG R:ACTAAGTTCACGAGCACCAGGAG
*mTOR*	XM_001788228.1	F:ATGCTGTCCCTGGTCCTTATG R:GGGTCAGAGAGTGGCCTTCAA
*EIF4EBP1*	BC120290.1	F:GAACTCACCTGTGACCAAGA R:CTCAAACTGTGACTCTTCACC
*EIF4E*	NM_174310.1	F:ACGAAGTGACCTCGATCGTT R:AGTAGCTGTGTCTGCATGGG
*S6K1*	NM_205816.1	F:CTGGGGAAGAGGTGCTTCAG R:GTGCTCTGGTCGTTTGGAGA
*RPS6*	NM_001010.2	F:AAGAGCTAGCAGAATCCGCA R:CGTGGAGTAACAAGACGCTG
*CSN1S1*	NM_181029.2	F:TCAACCCAGCTTGCTGCTTCTTCC R:GCCTAGCAAGAGCAACAGCCACAA
*CSN1S2*	NM_174528.2	F:AGCAGCTCTCCACCAGTGAGGAAA R:TGGGGCAAGGCGAATTTCTGGT
*β-actin*	NM_173979.3	F:GTCATCACCATCGGCAATGAG R:AATGCCGCAGGATTCCATG

**Table 6:** Antibody Information.

	Antibody	Source	Item Number	Dilution Methods	Dilution Ratio
Primary antibody	β-Actin	tdybio	TDY051	5% egg white	1:10,000
	P-PI3K	affbiotech	AF4372	5% egg white	1:1000
	PI3K	Proteintech	60225-1-Ig	5% egg white	1:2000
	P-AKT	Proteintech	66444-1-Ig	5% egg white	1:500
	AKT	Proteintech	60203-2-Ig	5% egg white	1:1000
	P-TSC2	bioss	bs-3442R	5% egg white	1:500
	TSC2	affbiotech	AF6334	5% egg white	1:1000
	P-mTOR	bioss	bs-3494R	5% egg white	1:500
	mTOR	bioss	bs-1992R	5% egg white	1:1000
	P-4EBP1	affbiotech	AF3432	5% egg white	1:1000
	4EBP1	affbiotech	AF6432	5% egg white	1:2000
	P-S6K1	affbiotech	AF3228	5% egg white	1:1000
	S6K1	affbiotech	AF6226	5% egg white	1:2000
	P-RPS6	affbiotech	AF3354	5% egg white	1:500
	RPS6	biorbyt	orb585017	5% egg white	1:1000
Secondary antibody	HRP-Goat anti-Rabbit	ASPEN	AS1107	5% skim milk	1:10,000
	HRP-Goat anti-Mouse	ASPEN	AS1106	5% skim milk	1:10,000
	HRP-Rabbit anti-Goat	ASPEN	AS1108	5% skim milk	1:10,000
	HRP-Goat anti-Rat	ASPEN	AS1093	5% skim milk	1:10,000

**Table 7:** Central Composite Design Matrix and Experimental Results for α-Casein Synthesis with Glycine, Asparagine, and Phenylalanine.

Run	A (Gly)	B (Asn)	C (Phe)	Y_1_ (α-Casein, μg/mL)
1	−1.000	−1.000	−1.000	191.415
2	1.000	−1.000	−1.000	170.611
3	−1.000	1.000	−1.000	186.65
4	1.000	1.000	−1.000	179.089
5	−1.000	−1.000	1.000	193.531
6	1.000	−1.000	1.000	194.093
7	−1.000	1.000	1.000	181.224
8	1.000	1.000	1.000	186.795
9	−2.000	0.000	0.000	179.39
10	2.000	0.000	0.000	170.159
11	0.000	−2.000	0.000	183.084
12	0.000	2.000	0.000	173.021
13	0.000	0.000	−2.000	179.656
14	0.000	0.000	2.000	190.399
15	0.000	0.000	0.000	200.805
16	0.000	0.000	0.000	196.23
17	0.000	0.000	0.000	192.182
18	0.000	0.000	0.000	196.603
19	0.000	0.000	0.000	197.305
20	0.000	0.000	0.000	196.27

A, B, and C represent the coded levels for glycine (Gly), asparagine (Asn), and phenylalanine (Phe), respectively, as defined in Table 3. Y_1_ represents the measured concentration of α-casein (μg/mL). Runs 15–20 are center points.

**Table 8:** Analysis of Variance (ANOVA) for the Response Surface Quadratic Model of α-Casein Synthesis.

Source	Sum of Squares	df	Mean Square	F-Value	*p*-Value
Model	1616.35	9	179.59	27.44	<0.0001
A-Glycine	103.50	1	103.50	15.82	0.0026
B-Asparagine	81.08	1	81.08	12.39	0.0055
C-Phenylalanine	152.30	1	152.30	23.27	0.0007
AB	41.64	1	41.64	6.36	0.0303
AC	148.76	1	148.76	22.73	0.0008
BC	67.97	1	67.97	10.39	0.0091
A^2^	701.55	1	701.55	107.20	<0.0001
B^2^	500.76	1	500.76	76.52	<0.0001
C^2^	185.89	1	185.89	28.40	0.0003
Residual	65.44	10	6.54		
Lack of fit	27.51	5	5.50	0.7250	0.6336
Pure error	37.94	5	7.59		
Total	1681.79	19			

**Table 9:** Model quality indicators for the response surface quadratic model of α-casein synthesis.

Parameter	Value	Parameter	Value
Std. Dev.	2.56	R-Squared	0.9611
Mean	186.93	Adj R-Squared	0.9261
C.V. %	1.37	Pred R-Squared	0.8429
		Adeq Precision	14.4927

## Data Availability

The data presented in this study are contained within the article.

## Supplementary Materials

The following supporting information can be downloaded at: https://www.mdpi.com/article/10.3390/ani16132038/s1, Figure S1: Melting curve analysis of qPCR products. The specificity of qPCR amplicons for ten target genes in MAC-T cells was verified by melting curve analysis. (A) *PI3K*; (B) *AKT1*; (C) *TSC2*; (D) *mTOR*; (E) *EIF4EBP1*; (F) *EIF4E*; (G) *S6K1*; (H) *RPS6*; (I) *CSN1S1*; (J) *CSN1S2*. All melting curves displayed a single specific melting peak (distinct Tm value) without detectable primer-dimer peaks or non-specific products, confirming the high specificity of each primer pair.

## Abbreviations

The following abbreviations are used in this manuscript:

MAC-T	Bovine mammary epithelial cell line
CCD	Central composite design
ELISA	Enzyme-linked immunosorbent assay
PI3K	Phosphoinositide 3-kinase
AKT	Protein kinase B
mTOR	Mammalian target of rapamycin
DMEM	Dulbecco’s Modified Eagle Medium
FBS	Fetal bovine serum
DMSO	Dimethyl sulfoxide
