# Supplementary material for: Optimal Combination of Glycine, Asparagine, and Phenylalanine Promotes α-Casein Synthesis and Secretion in MAC-T Cells Through Activation of the PI3K-AKT-mTOR Pathway
Source: Animals (Basel). 2026 Jul 2;16(13):2038. doi: 10.3390/ani16132038 (PMC13359911; doi:10.3390/ani16132038)
Supplement: Supplementary file 1 [file animals-16-02038-s001.zip › animals-4348580-supplementary.pdf]

Supplementary: Melting Curve Analysis of qPCR Products

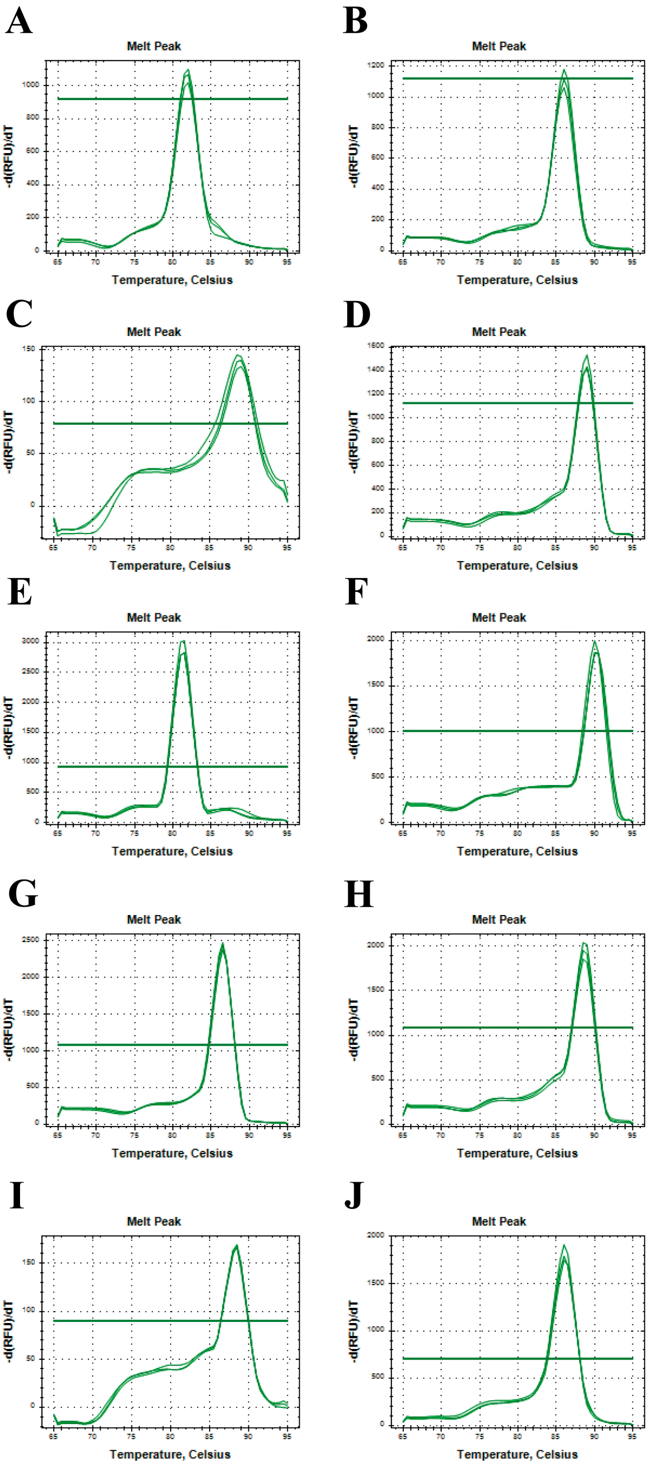

Figure S1. Melting curve analysis of qPCR products. The specificity of qPCR amplicons for ten target genes in MAC-T cells was verified by melting curve analysis. (A) PI3K; (B) AKT1; (C) TSC2; (D) mTOR; (E) EIF4EBP1; (F) EIF4E; (G) S6K1; (H) RPS6; (I) CSN1S1; (J) CSN1S2. All melting curves displayed a single specific melting peak (distinct Tm value) without detectable primer-dimer peaks or non-specific products, confirming the high specificity of each primer pair.
